# The Role of T_H_17-Associated Cytokines in Health and Disease

**DOI:** 10.1155/2014/936270

**Published:** 2014-04-29

**Authors:** William O'Connor, Enric Esplugues, Samuel Huber

**Affiliations:** ^1^Center for Immunology and Microbial Disease, Albany Medical College, Albany, NY 12208, USA; ^2^Immunology Institute, Mount Sinai School of Medicine, Icahn Medical Institute, 1425 Madison Avenue, New York, NY 10029, USA; ^3^Medical Department, University Medical Center Hamburg-Eppendorf, Martinistraße 52, 20246 Hamburg, Germany

The intriguing subset of effector CD4+ T cells termed T_H_17 cells are now widely appreciated for their role in coordinating immune and inflammatory responses. The dynamic nature of the T_H_17 cell subset allows for the adoption of inflammatory or regulatory functions as needed, in a microenvironment-dependent fashion. The ontogeny, tissue residence, migratory properties, and biological functions of these cells are areas of intense research focus given the broad spectrum of human disorders associated with aberrant T_H_17-type responses.

While T_H_17 cells are so named for their characteristic Interleukin 17 (IL-17) production,* bona fide *T_H_17 cells of human or murine origin produce, at times, a cacophony of inflammatory mediators, which can include IL-17, IL-21, IL-22, and IL-26. As such, dissecting the biological consequences of robust T_H_17 responses, properly or improperly controlled, has presented a number of challenges. Further confounding the study of T_H_17 cells and individual cytokines are the many observations documenting non- T_H_17 cell sources of these same cytokines. Given the complexity of T_H_17 biology, we welcome the reports found within this issue, highlighting current findings and observations and illuminating several components of T_H_17 cells and known associated cytokines.

N. Qu et al. provide an interesting overview of the roles T_H_17 cells and their associated cytokines play in various inflammatory diseases. N. Y. A. Hemdan et al. build on this premise, addressing the T_H_17 cell contributions to autoimmunity, in particular that which arises following exposure to xenobiotic substances.

In the absence of overt chronic inflammation, T_H_17 cells predominantly reside at mucosal surfaces. H.-C. Tsai et al. provide an elegant update on the functions of T_H_17 differentiation and on the functional consequences of IL-17 signaling in pulmonary inflammation. Y. Morishima et al. highlight the role of T_H_17-associated cytokines in asthma, especially in steroid-resistant disease. Further interesting findings from the Hizawa laboratory suggest an important role for IL-17F in particular.

T_H_17 cells, which are known to be induced in response to a variety of bacterial and fungal infections, may also be selectively depleted, as in the early stages of an HIV infection. S. L. Bixler and J. J. Mattapallil elegantly discuss potential mechanisms by which T_H_17 cells are depleted or improperly regulated during HIV infection.

Further, T_H_17 cells and T_H_17-produced cytokines have also been associated with tumor immunity and conversely with promoting the initiation/progression of tumorigenesis. In this issue, D. Alizadeh et al. discuss how T_H_17 cells and T_H_17-associated cytokines may act directly or indirectly toward shifting local microenvironments to favor tumor promotion or tumor suppression. Focusing on AML, T. Tian et al. examined T_H_17 cell frequencies in acute myeloid leukemia patients and discuss their observed stage-dependent variation.

It is our hope that you will find the articles within insightful; we have enjoyed reading all of these articles immensely.

*William O'Connor Jr.* *William O'Connor Jr.* *Enric Esplugues* *Enric Esplugues* *Samuel Huber* *Samuel Huber*

